# Refined Self-Motion Scheme With Zero Initial Velocities and Time-Varying Physical Limits *via* Zhang Neurodynamics Equivalency

**DOI:** 10.3389/fnbot.2022.945346

**Published:** 2022-08-18

**Authors:** Zanyu Tang, Yunong Zhang

**Affiliations:** ^1^School of Computer Science and Engineering, Sun Yat-sen University, Guangzhou, China; ^2^Research Institute of Sun Yat-sen University in Shenzhen, Shenzhen, China; ^3^College of Information Science and Engineering, Jishou University, Jishou, China

**Keywords:** self-motion control scheme, zero initial joint-angle velocities, time-varying physical limits, Zhang neurodynamics equivalency, redundant robot manipulators, quadratic program

## Abstract

By considering the different-level time-varying physical limits in joint space, a refined self-motion control scheme *via* Zhang neurodynamics equivalency (SMCSvZ) of redundant robot manipulators is proposed, analyzed, and investigated in this manuscript. The SMCSvZ is reformulated as a quadratic program with an equation constraint and a unified bound inequation constraint, which meets the self-motion requirements including the end effector keeping immobile and the initial joint-angle velocities being zero. Simulative verifications based on a six-degrees-of-freedom planar redundant manipulator substantiate the efficacy, accuracy, and superiority of the proposed control scheme, additionally by comparing it with two previous self-motion control schemes. Besides, simulative verifications based on a PUMA560 manipulator are carried out to further verify the availability and correctness of the SMCSvZ.

## 1. Introduction

Redundant robot manipulators refer to such kind of manipulators whose degrees of freedom (DoF) are more than the minimum number of DoF needed to perform specific end-effector tasks (Zhang et al., 2018; Liao et al., 2019; Zhou et al., 2019; Chen et al., 2020; Xiao et al., 2020; Zhao et al., 2020; Jin et al., 2021). Therefore, they have the capability to meet additional requirements, e.g., satisfying physical limits, avoiding obstacles, and avoiding singularity configurations. In the practical application, the redundant robot manipulator needs to adjust its configuration in some peculiar situations. For instance, the repetitive motion of the redundant robot manipulator is planned but joint-angle drift may happen. Similarly, the end-effector task may not be completed because of operating space limitations or manipulator physical limitations. Adjusting the manipulator configuration from one state to another state is essential and important for redundant robot manipulators (Jin et al., 2020). Thereinto, the self-motion of redundant robot manipulators is to adjust the manipulator configuration from the initial state to final state keeping the end effector immobile at its current position or orientation (Li and Zhang, 2012; Zhang et al., 2021a). The self-motion could result in better manipulator performance such as manipulability improvement (Jin et al., 2021), end-effector task completeness, and singularity configuration avoidance (Pardi et al., 2020).

In recent years, many self-motion control schemes (SMCSs) or self-motion control approaches have been developed (Zhang et al., 2009, 2016, 2021a; Li and Zhang, 2012; Zhang and Xiao, 2012; Gong et al., 2019). For instance, a self-motion scheme in form of a quadratic program (QP) was presented in Zhang et al. (2009) and Liao et al. (2021), which was verified on the functionally redundant robot manipulator PUMA560 considering joint-angle limits and joint-angle-velocity limits. With singularities discussed, Zhang and Xiao (2012) proposed a QP-based self-motion scheme for manipulators compared with the pseudoinverse method and substantiated that the proposed scheme was effective on three kinds of manipulators. To eliminate the abrupt increase in joint velocity at the beginning of the self-motion task execution, Li and Zhang (2012) put forward a zero-initial-velocity self-motion scheme and verified its feasibility on a 6-DoF planar manipulator. Besides, in order to achieve high efficient self-motion tasks, Zhang et al. (2021a) put forth a varying-gain neural self-motion approach.

In many previous studies, researchers developed QP-based SMCSs considering time-invariant physical limits. However, some redundant manipulators are inherently subject to varying physical limits (Li and Zhang, 2012). Besides, in the engineering field, with the passage of time and mechanical wear, the physical limits may change with time, i.e., they are time-varying. Considering this case, we establish a refined SMCS for the redundant manipulators *via* the equivalency method in this article.

Zhang neurodynamics equivalency (ZNDE) inspired by Ma equivalency (Ma, 1996; Ma et al., 1996), is actually a class of practice-accepted approximation, which is derived from Zhang neurodynamics (Chen and Zhang, 2018; Qin et al., 2021). Some schemes of complex systems *via* the ZNDE approach were efficiently simplified (Guo et al., 2013; Qiu et al., 2016, 2018). Minimum-velocity-norm schemes of redundant robot manipulators at two different layers were established by Guo et al. (2013), and the equivalent relationship between two manipulator control schemes was also developed *via* the Zhang neurodynamics method. Zhang et al. (2020b) substantiated that the schemes of redundant robot manipulators formulated by the ZNDE approach were more robust. Zhang et al. (2020a) tried to solve complex inequality-related problems through the ZNDE approach. Besides, Zhang et al. (2021b) proposed a cyclic motion control scheme at the acceleration layer for manipulator systems *via* the ZNDE approach.

In this article, a refined self-motion control scheme *via* the ZNDE approach (named SMCSvZ) in form of standard QP is proposed to solve self-motion problems. With the time-varying physical limits considered and zero initial joint-angle velocities ensured, the proposed SMCSvZ is developed, proved, and obtained by the ZNDE theorem and the corresponding corollary. The simulation experiments based on two different robot manipulators are designed and carried out to substantiate the correctness and superiority of the proposed scheme by comparing the previous SMCSs. The remainder of this article consists of five sections. In Section 2, the requirements of self-motion problems are presented first. Then, by analyzing and comparing the two previous SMCSs, the refined SMCSvZ is proposed *via* the ZNDE approach. In Section 3, the derivation process of the SMCSvZ is provided, and the feasibility and availability of the SMCSvZ are analyzed theoretically. In Section 4, the SMCSvZ composed of performance index, equivalent equation constraint, and bounded inequality constraint is presented in form of standard QP formulation, and its corresponding neural network solver is also shown. In Section 5, the simulation experiments based on a 6-DoF planar manipulator are carried out, and the simulation results substantiate the efficacy and superiority of the SMCSvZ. Moreover, the simulation experiments based on a PUMA560 manipulator are also carried out to further verify the availability and correctness of the SMCSvZ. Finally, we conclude the paper in Section 6. The main contributions of the current study are presented as follows.

1. To better meet self-motion requirements, a refined self-motion control scheme of redundant robot manipulators is proposed with time-varying physical limits and zero initial joint-angle velocities considered.
2. The theorem and corollary of the ZNDE approach are proposed and theoretically proved, through which the refined SMCSvZ is obtained. Then, the SMCSvZ is applied to redundant manipulators to effectively realize the self-motion task.
3. By comparing the SMCSvZ with the two previous SMCSs, the simulation experiments based on a 6-DoF planar redundant manipulator and a PUMA560 manipulator are carried out with physical limits fully satisfied, which verifies the availability, effectiveness, and superiority of the proposed SMCSvZ.

## 2. Preliminary, Problem, and Schemes

The forward-kinematics equation of redundant robot manipulators is written as **r** = *Ϝ*(Θ), where **r** ∈ ℝ^*m*^ denotes the end-effector actual position with Θ ∈ ℝ^*n*^ denoting the joint-angle vector and *Ϝ*(·) being a differentiable nonlinear function. Furthermore, the inverse-kinematics equation about relationship between the derivative of end-effector position vector $\dot{\text{r}}\in {\mathbb{R}}^{m}$ and the derivative of joint-angle-velocity vector $\dot{\Theta }\in {\mathbb{R}}^{n}$ is written as


$$\begin{array}{l} J\left(\Theta \right)\dot{\Theta }=\dot{\text{r}}, \end{array}$$

where *J*(Θ) = ∂*Ϝ*(Θ)/∂Θ ∈ ℝ^*m* × *n*^ is the Jacobian matrix.

In essence, the self-motion task of the redundant robot manipulator is to utilize the redundant DoF of the manipulator to adjust its configuration in joint space with the end effector being immobile. For guaranteeing the end effector is immobile, the self-motion task can be completed with the given joint angles being in the motion region. If the given joint angles are out of the motion region, the redundant robot manipulators also try to adjust the configuration to a suitable state. As a result, the manipulators become more flexible after the self-motion task. To execute the self-motion task, the SMCS of redundant robot manipulators needs to meet the following requirements, which constitute the problem formulation. ① Robot manipulators adjust joint angles Θ(0) to given joint angles Θ_g_ that are suitable joint angles within the workspace of redundant robots (Akli, 2021). ② Robot manipulators try to keep the end effector immobile during the process of self motion. ③ The initial velocities of the joint angles equal zero. ④ The final velocities of the joint angles equal zero. ⑤ Time-varying physical limits (including joint-angle layer and joint-angle-velocity layer limits) of redundant robot manipulators are all satisfied. Accordingly, we depict those requirements as


(1) $$\begin{array}{l} \Theta \left(t\right)\to \Theta_{\text{g}}, \end{array}$$

(2) $$\begin{array}{l} Ϝ\left(\Theta \left(t\right)\right)\to Ϝ\left(\Theta \left(0\right)\right), \end{array}$$

(3) $$\begin{array}{l} \dot{\Theta }\left(0\right)=0, \end{array}$$

(4) $$\begin{array}{l} \dot{\Theta }\left(t_{\text{end}}\right)=0,\text{ with }t_{\text{end}}\in \left[0,t_{\text{f}}\right] \end{array}$$

(5) $$\begin{array}{l} {\text{l}}_{0}^{-}\left(t\right)\leq \Theta \left(t\right)\leq {\text{l}}_{0}^{+}\left(t\right), \end{array}$$

(6) $$\begin{array}{l} {\text{l}}_{1}^{-}\left(t\right)\leq \dot{\Theta }\left(t\right)\leq {\text{l}}_{1}^{+}\left(t\right), \end{array}$$

where Θ(*t*) ∈ ℝ^*n*^ and $\dot{\Theta }\left(t\right)\in {\mathbb{R}}^{n}$ denote the joint-angle vector and joint-angle-velocity vector, respectively; Θ_g_ is the given joint-angle vector; Θ(0) is the initial joint-angle vector; $\dot{\Theta }\left(t\right)$ denotes the derivative of Θ(*t*) with time instant *t* ∈ [0, *t*_f_] and *t*_f_ denoting last instant time of the self-motion duration; $\dot{\Theta }\left(0\right)$ denotes $\dot{\Theta }\left(t\right)$ with *t* = 0; $\dot{\Theta }\left(t_{\text{end}}\right)$ denotes $\dot{\Theta }\left(t\right)$ with *t*_end_ being the end time of the self-motion task. In addition, ${\text{l}}_{0}^{-}\left(t\right)$ and ${\text{l}}_{0}^{+}\left(t\right)$ represent the time-varying joint-angle lower limit and upper limit, respectively; ${\text{l}}_{1}^{-}\left(t\right)$ and ${\text{l}}_{1}^{+}\left(t\right)$ represent the joint-angle-velocity lower limit and upper limit, respectively. The traditional SMCS at the joint-angle-velocity layer is formulated in Zhang et al. (2009) as


(7) $$\begin{array}{ll} \text{minimize} & \|\dot{\Theta }\left(t\right)+\text{q}\left(t\right){\|}_{2}^{2}/2, \end{array}$$

(8) $$\begin{array}{ll} \text{subject to} & J\left(\Theta \left(t\right)\right)\dot{\Theta }\left(t\right)=0, \end{array}$$

(9) l1 −≤Θ.(t)≤l1 +,

(10) $$\begin{array}{ll} \text{with} & \text{q}\left(t\right)=\mu \left(\Theta \left(t\right)-\Theta \text{g}\right), \end{array}$$

(11) l1 −=max{κ(l0−−Θ(t)),l1−},

(12) l1 +=min{κ(l0+−Θ(t)),l1+},

where symbol ‖·‖_2_ denotes the two-norm of the vector, and the time-varying vector **q**(*t*) ∈ ℝ^*n*^ is defined according to the self-motion task. The design parameters μ > 0 ∈ ℝ and κ > 0 ∈ ℝ are used to scale the magnitude of the manipulators. The “max” and “min” functions are used to obtain the maximum and minimum values of elements in the vector, respectively. The other parameters are the same as those of the requirements (1)–(6). We name the scheme (7)–(12) as SMCS-1 in this article.

As a further research of Zhang et al. (2009), a zero-initial-velocity self-motion scheme for redundant robot manipulators is proposed in Li and Zhang (2012) as shown below:


(13) $$\begin{array}{ll} \text{minimize} & \|\dot{\Theta }\left(t\right)+\text{q}\left(t\right){\|}_{2}^{2}/2,\; \end{array}$$

(14) $$\begin{array}{ll} \text{subject to} & J\left(\Theta \left(t\right)\right)\dot{\Theta }\left(t\right)=0,\; \end{array}$$

(15) l2 −≤Θ.(t)≤l2 +, 

(16) $$\begin{array}{ll} \text{with} & \text{q}\left(t\right)=\mu \left(\Theta \left(t\right)-\Theta \text{g}\right),\; \end{array}$$

(17) l2 −=max{κ(l0−−Θ(t)),sin(πt/(2tf))l1−}, 

(18) l2 +=min{κ(l0+−Θ(t)),sin(πt/(2tf))l1+}, 

where the physical limits (^2^**l**^+^ and ^2^**l**^−^) are partly different from ^1^**l**^±^ presented in (17) and (18), which ensure $\dot{\Theta }\left(0\right)=0$. The other parameters are the same as those of SMCS-1. The scheme (13)–(18) is named SMCS-2 in this article.

However, equation limits (8) in SMCS-1 and (14) in SMCS-2 are difficult to realize in practice. Different from SMCS-1 and SMCS-2, with comprehensive consideration of continuously and differentially time-varying physical limits, zeroing initial joint-angle velocities, and dynamically keeping the end-effector position immobile, we propose a refined SMCSvZ for redundant robot manipulators in this article, which is formulated as


(19) $$\begin{array}{ll} \text{minimize} & \|\dot{\Theta }\left(t\right)+\text{q}\left(t\right){\|}_{2}^{2}/2,\; \end{array}$$

(20) $$\begin{array}{ll} \text{subject to} & J\left(\Theta \left(t\right)\right)\dot{\Theta }\left(t\right)=-\mu_{1}\left(Ϝ\left(\Theta \left(t\right)\right)-Ϝ\left(\Theta \left(0\right)\right)\right),\; \end{array}$$

(21) l3 −≤Θ.≤l3 +, 

(22) $$\begin{array}{ll} \text{with} & \text{q}\left(t\right)=\mu_{2}t\left(\Theta \left(t\right)-\Theta \text{g}\right),\; \end{array}$$

(23) l3 −(t)=max{l˙0−(t)+κ(l0−(t)−Θ(t)),l1−(t)}, 

(24) l3 +(t)=max{l˙0+(t)+κ(l0+(t)−Θ(t)),l1+(t)},

where the positive design parameters μ_1_, μ_2_, and κ are used to scale the magnitude of the manipulators. In addition, ${\text{l}}_{0}^{-}\left(t\right)$, ${\text{l}}_{0}^{+}\left(t\right)$, ${\text{l}}_{1}^{-}\left(t\right)$, and ${\text{l}}_{1}^{+}\left(t\right)$ are the same as those of SMCS-1; ${\dot{\text{l}}}_{0}^{-}\left(t\right)$ and ${\dot{\text{l}}}_{0}^{+}\left(t\right)$ represent the derivatives of ${\text{l}}_{0}^{-}\left(t\right)$ and ${\text{l}}_{0}^{+}\left(t\right)$, respectively.

## 3. SMCSvZ Derivation and Analysis

In this section, the performance index, the equation constraint, and the unified bound inequation constraint in the QP-based SMCSvZ are deduced *via* the ZNDE approach. The theorem and corollary are given and proved for the analysis of the SMCSvZ.

### 3.1. Equation Constraint *via* ZNDE

In this subsection, the equivalence analyses of equations in SMCSvZ are carried out. To be specific, (19), (20), and (22) in SMCSvZ are derived and analyzed theoretically.

To ensure that initial joint-angle velocities are zero, physical limits (^2^**l**^±^) in SMCS-2 are different from ^1^**l**^±^ in SMCS-1, and one part of ^2^**l**^±^ is obtained through multiplying sin(π*t*/(2t_f_)) by ^1^**l**^±^. These changes realize $\dot{\Theta }\left(0\right)=0$ but reduce the feasible region of $\dot{\Theta }\left(t\right)$. That is when the physical limits verge, $\dot{\Theta }\left(t\right)$ change to avoid exceeding the physical limits, but they can only change slowly and thus make the task spend more time. In SMCSvZ, we define (22) instead of (10) because the Equation (22) can better meet the requirements of self-motion tasks and it is practically and mathematically equivalent to Equation (10), which is proved *via* the following ZNDE-EEV theorem.

**Theorem 1**. *(ZNDE-EEV theorem) With differentiable **ϵ**(*t*) ∈ ℝ^*n*^, the zero vector **0** ∈ ℝ^*n*^, sufficiently large positive design parameter μ ≫ 0 and time instant *t* ≫ 0*,


(25) $$\begin{array}{l} \dot{\epsilon }\left(t\right)=-\mu t\epsilon \left(t\right) \end{array}$$

*is practically mathematically equivalent (i.e., ZNDE equivalent) to*


(26) $$\begin{array}{l} \epsilon \left(t\right)=0. \end{array}$$

*Proof:* The Equation (25) is a differential equation with $\dot{\epsilon }\left(t\right)$ denoting d(**ϵ**(*t*))/d*t*. The analytical solution of (25) is **ϵ**(*t*) = **ϵ**(0)exp(−μ*t*^2^/2) with **ϵ**(0) denoting the initial value of **ϵ**(*t*) and μ denoting a large positive design parameter.

With *t* → ∞, **ϵ**(*t*) = **ϵ**(0)exp(−μ*t*^2^/2) → **0** instantaneously. That is, each element of **ϵ**(*t*) quickly decreases to a tiny value that is considered to be zero in practical application, or **ϵ**(*t*) = **0** with *t* large enough. Therefore, (25) is ZNDE equivalent to (26) with μ ≫ 0 and *t* ≫ 0. The proof, thus, ends.      ■

To settle the self-motion problem of redundant robot manipulators, we combine the self-motion requirement (1) and define an error function as **ϵ**(*t*) = Θ(*t*) − Θ_g_. Then, according to Theorem 1, one gets that


(27) $$\begin{array}{l} \dot{\epsilon }\left(t\right)=\dot{\Theta }\left(t\right)=-\mu t\left(\Theta \left(t\right)-\Theta_{\text{g}}\right) \end{array}$$

is ZNDE equivalent to Θ(*t*) − Θ_g_ = **0**. From (27), one can get the following. First, the performance index at the velocity layer can be expressed as $\|\dot{\Theta }\left(t\right)+\mu t\left(\Theta \left(t\right)-\Theta_{\text{g}}\right){\|}_{2}^{2}/2$, i.e., **q**(*t*) = μ*t*(Θ(*t*) − Θ_g_), which corresponds to (19) and (22) in SMCSvZ. Second, the self-motion task ends when the value of Θ(*t*_end_) − Θ_g_ equals **0** with *t*_end_ ∈ [0, *t*_f_]. That is when the final velocities of joint angles $\dot{\Theta }\left(t_{\text{end}}\right)$ verge or equal **0**, the self-motion task is considered to be over. Finally, when *t* = 0, the initial joint-angles velocity vector $\dot{\Theta }\left(0\right)$ equals **0**. The above analyses show that (22) in SMCSvZ obtained by the ZNDE better meets the requirements (1), (3), and (4) of the self-motion task.

In addition, the self-motion requirement (2) is formulated as $J\left(\Theta \left(t\right)\right)\dot{\Theta }\left(t\right)=0$ in SMCS-1 and SMCS-2, which is difficult to guarantee in practice. Hence, we handle the problem by transforming this constraint to (20) in SMCSvZ *via* the ZNDE approach, which is illustrated clearly by the following lemma (Zhang et al., 2021b).

**Lemma 1**. *With differentiable **ϵ**(*t*) ∈ ℝ^*n*^, the zero vector **0** ∈ ℝ^*n*^, sufficiently large positive design parameter μ ≫ 0 and time instant *t* ≫ 0, $\dot{\epsilon }\left(t\right)=-\mu \epsilon \left(t\right)$ is practically mathematically equivalent (i.e., ZNDE equivalent) to **ϵ**(*t*) = **0***.

To meet the self-motion requirement (2), the error function is defined as **ϵ**(*t*) = *Ϝ*(Θ(*t*)) − *Ϝ*(Θ(0)). By Lemma 1, we get that the equation $J\left(\Theta \right)\dot{\Theta }\left(t\right)=-\mu \left(Ϝ\left(\Theta \left(t\right)\right)-Ϝ\left(\Theta \left(0\right)\right)\right)$ is ZNDE equivalent to (8) in SMCS-1 and (14) in SMCS-2, which is just equation constraint (20) in SMCSvZ. Meanwhile, equivalent equation constraint (20) in SMCSvZ dynamically keeps the end effector nearest to its initial position.

### 3.2. Inequation Constraint *via* ZNDE

In this subsection, we are to unify two-layer inequation constraints into one equivalent bound inequation constraint through the inequation type of the ZNDE that is described in the following lemma (Zhang et al., 2021b).

**Lemma 2**. *With differentiable **ϵ**(*t*) ∈ ℝ^*n*^, sufficiently large positive design parameter ρ ≫ 0 and time instant *t* ≫ 0, in a ZND manner, $\dot{\epsilon }\left(t\right)\leq -\rho \epsilon \left(t\right)$ is practically mathematically equivalent (i.e., ZNDE equivalent) to **ϵ**(*t*) ≤ **0***.

According to Lemma 2, the following corollary at the velocity layer is acquired.

**Corollary 1**. *Assume that vector Θ(*t*) and its time-varying physical limits ${\text{l}}_{0}^{\pm }\left(t\right)$ are continuously differentiable. ${\dot{\text{l}}}_{0}^{-}\left(t\right)$ and ${\dot{\text{l}}}_{0}^{+}\left(t\right)$ represent the derivatives of ${\text{l}}_{0}^{-}\left(t\right)$ and ${\text{l}}_{0}^{+}\left(t\right)$, respectively. With design parameter ρ ≫ 0 and time *t* ≫ 0, in a ZND manner*,


(28) $$\begin{array}{l} {\dot{\text{l}}}_{0}^{-}\left(t\right)-\rho \left(\Theta \left(t\right)-{\text{l}}_{0}^{-}\left(t\right)\right)\leq \dot{\Theta }\left(t\right)\leq {\dot{\text{l}}}_{0}^{+}\left(t\right)-\rho \left(\Theta \left(t\right)-{\text{l}}_{0}^{+}\left(t\right)\right) \end{array}$$

*is practically mathematically equivalent (i.e., ZNDE equivalent) to*


(29) $$\begin{array}{l} {\text{l}}_{0}^{-}\left(t\right)\leq \Theta \left(t\right)\leq {\text{l}}_{0}^{+}\left(t\right). \end{array}$$

*Proof:* By defining the function $\epsilon \left(t\right)=\Theta \left(t\right)-{\text{l}}_{0}^{+}\left(t\right)\leq 0$ according the left part of (29), one gets $\dot{\epsilon }\left(t\right)=\dot{\Theta }\left(t\right)-{\dot{\text{l}}}_{0}^{+}\left(t\right)\leq -\rho \left(\Theta \left(t\right)-{\text{l}}_{0}^{+}\left(t\right)\right)$, which is ZNDE equivalent to $\Theta \left(t\right)\leq {\text{l}}_{0}^{+}\left(t\right)$ *via* Lemma 2. Then, $\dot{\Theta }\left(t\right)\leq {\dot{\text{l}}}_{0}^{+}\left(t\right)-\rho \left(\Theta \left(t\right)-{\text{l}}_{0}^{+}\left(t\right)\right)$ is obtained.

Similarly, by defining the function $\epsilon \left(t\right)={\text{l}}_{0}^{-}\left(t\right)-\Theta \left(t\right)\leq 0$ according the right part of (29), one gets $\dot{\epsilon }\left(t\right)={\dot{\text{l}}}_{0}^{-}\left(t\right)-\dot{\Theta }\left(t\right)\leq -\rho \left({\text{l}}_{0}^{-}\left(t\right)-\Theta \left(t\right)\right)$, which is ZNDE equivalent to ${\text{l}}_{0}^{-}\left(t\right)\leq \Theta \left(t\right)$ *via* Lemma 2. Then, ${\dot{\text{l}}}_{0}^{-}\left(t\right)+\rho \left({\text{l}}_{0}^{-}\left(t\right)-\Theta \left(t\right)\right)\leq \dot{\Theta }\left(t\right)$ is obtained. Combined with the above results, the corollary is proved.      ■

From the above corollary, (28) is ZNDE equivalent to the self-motion requirement (5). By combining (28) and the self-motion requirement (6), the unified equivalent bound inequation constraint (21) in SMCSvZ is obtained.

## 4. QP Formulation and Projection Neural Network (PNN) Solver

By using the ZNDE approach, the QP-based SMCSvZ is obtained to control the redundant robot manipulators for realizing the self-motion task, which is handled by a projection neural network (PNN) solver.

### 4.1. Standard QP Formulation

On the basis of Theorem 1, Lemma 1, and Corollary 1, the SMCSvZ with time-varying physical limits satisfied is reformulated as a standard QP at the velocity layer as follows.


(30) $$\begin{array}{ll} \text{minimize} & \frac{1}{2}\Upsilon^{\text{T}}\left(t\right)A\left(t\right)\Upsilon \left(t\right)+{\text{p}}^{\text{T}}\left(t\right)\Upsilon \left(t\right),\; \end{array}$$

(31) $$\begin{array}{ll} \text{subject to} & B\left(t\right)\Upsilon \left(t\right)=\text{b}\left(t\right),\; \end{array}$$

(32) $$\begin{array}{l} {\text{l}}^{-}\left(t\right)\leq \Upsilon \left(t\right)\leq {\text{l}}^{+}\left(t\right),\; \end{array}$$

(33) $$\begin{array}{ll} \text{with} & {\text{l}}^{-}\left(t\right)=\max\left\{\overset{-}{\underset{0}{\dot{\text{l}}}}\left(t\right)+\kappa \left({\text{l}}_{0}^{-}\left(t\right)-\Theta \left(t\right)\right),{\text{l}}_{1}^{-}\left(t\right)\right\},\; \end{array}$$

(34) $$\begin{array}{ll} \; & {\text{l}}^{+}\left(t\right)=\min\left\{\overset{+}{\underset{0}{\dot{\text{l}}}}\left(t\right)+\kappa \left({\text{l}}_{0}^{+}\left(t\right)-\Theta \left(t\right)\right),{\text{l}}_{1}^{+}\left(t\right)\right\},\; \end{array}$$

where $\Upsilon \left(t\right)=\dot{\Theta }\left(t\right)$; *A*(*t*) = *I*_*n*_ denotes an *n* × *n* identity matrix; *B*(*t*) = *J*(Θ(*t*)); **p**(*t*) = μ_1_*t*(Θ(*t*) − Θ(0)); **b**(*t*) = − μ_2_(*Ϝ*(Θ(*t*)) − *Ϝ*(Θ(0))) with μ_1_ and μ_2_ presenting the design parameters. Moreover, **l**^+^(*t*) and **l**^−^(*t*) are the physical upper and lower limits of synthesized time-varying unified layer, respectively. ${\text{l}}_{0}^{-}\left(t\right)$, ${\text{l}}_{0}^{+}\left(t\right)$, ${\text{l}}_{1}^{-}\left(t\right)$, ${\text{l}}_{0}^{+}\left(t\right)$, ${\dot{\text{l}}}_{0}^{-}\left(t\right)$, and ${\dot{\text{l}}}_{0}^{+}\left(t\right)$ are the same as above SMCSvZ (19)–(24).

### 4.2. PNN Solver

To solve the QP-based SMCSvZ (30)–(34) in real time, we use a PNN solver to obtain the solution **Υ**(*t*) in this subsection, which is developed in the following lemma (Xia et al., 2020).

**Lemma 3**. *With γ ∈ ℝ^+^ adjusting the convergence rate and large enough ς, the PNN solver for SMCSvZ is developed as*


(35) $$\begin{array}{l} \dot{u}(t)=\gamma (I_{n+m}+M^{\text{T}}(t))(P_{\Omega }(u(t)-(M(t)u(t)+h(t)))-u(t)), \end{array}$$

*where **u**(*t*) = [**Υ**(*t*);**ϖ**] ∈ ℝ^*n*+*m*^ and **h**(*t*) = [**p**(*t*);−**b**(*t*)] ∈ ℝ^*n*+*m*^ in MATLAB manner (Mathews and Fink, 2004). Meanwhile, *I*_*n*+*m*_ denotes a (*n* + *m*) × (*n* + *m*) identity matrix, and **ϖ** ∈ ℝ^*m*^ is the dual decision vector defined corresponding to (31). Besides*,


$$\begin{array}{l} M\left(t\right)=\left[\begin{array}{cc} A\left(t\right) & -B^{\text{T}}\left(t\right)\\ B\left(t\right) & O_{m} \end{array}\right]\in {\mathbb{R}}^{\left(n+m\right)\times \left(n+m\right)}, \end{array}$$

$$\begin{array}{l} {\text{u}}^{-}\left(t\right)=\left[\begin{array}{c} {\text{l}}^{-}\left(t\right)\\ -\varsigma 1_{\text{v}} \end{array}\right]\in {\mathbb{R}}^{n+m},\text{and }{\text{u}}^{+}\left(t\right)=\left[\begin{array}{c} {\text{l}}^{+}\left(t\right)\\ \varsigma 1_{\text{v}} \end{array}\right]\in {\mathbb{R}}^{n+m}, \end{array}$$

*in which *O*_*m*_ denotes an *m* × *m* zero matrix and $1_{\text{v}}={\left[1,\cdots \;,1\right]}^{\text{T}}\in {\mathbb{R}}^{m}$*.

## 5. Simulations and Comparisons

In this section, the simulation experiments are conducted based on two different redundant robot manipulators, which include a 6-DoF planar manipulator and a PUMA560 manipulator. Thereinto, the PUMA560 manipulator works in three-dimensional space.

### 5.1. Simulations Based on 6-DoF Planar Manipulator

In the ensuing simulations, the initial joint states of the planar manipulator are set as [0.628, 1.047, −1.570, 1.570, −0.785, 1.047]^T^ rad with superscript ^T^ denoting the transposition of the vector, and the given joint states are set as [1.574, 0.129, −0.947, 1.091, −1.928, 1.067]^T^ rad. The task time-interval is set as [0, 3] s.

#### 5.1.1. Case A: Loose Physical Limits

First, the simulation experiments are conducted with all physical limits satisfied. The joint-angle limits and joint-angle-velocity limits are time-varying, and the limits region are set loose. Specifically, each element in ${\text{l}}_{0}^{-}\left(t\right)$ is set as −3+0.25sin^2^(*t*) rad, and each element in ${\text{l}}_{0}^{+}\left(t\right)$ is set as 3−0.25sin^2^(2*t*) rad. Each element in ${\text{l}}_{1}^{-}\left(t\right)$ is set as −3+0.25sin^2^(2*t*) rad/s, and each element in ${\text{l}}_{1}^{+}\left(t\right)$ is set as 3−0.25sin^2^(2*t*) rad/s. The corresponding parameters are set as γ = 10^4^, ς = 10^6^, and μ_1_ = μ_2_ = 3.

**Figure 1:**
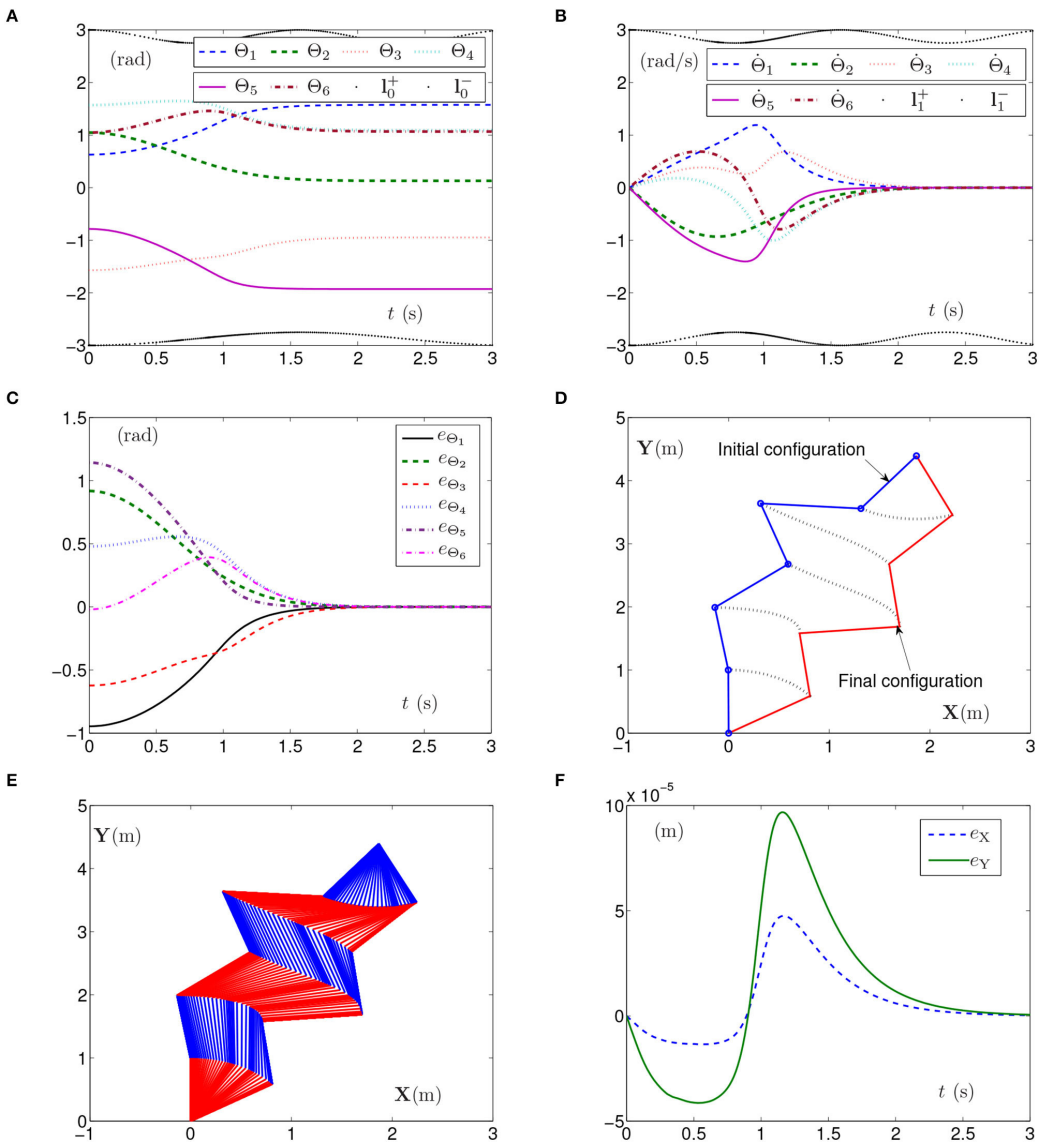
Synthesized results of the planar manipulator using SMCSvZ with time-varying physical limits satisfied in case A. **(A)** Profiles of joint angles. **(B)** Profiles of joint-angle velocities. **(C)** Profiles of joint-angle errors. **(D)** Profiles of initial and final manipulator positions. **(E)** Profiles of the planar manipulator. **(F)** Profiles of end-effector position errors.

By PNN solver, the simulation results synthesized by the planar manipulator using the SMCSvZ are generated and presented in Figure 1. The curves of joint angles with the time-varying physical limits satisfied are presented in Figure 1A. As seen from Figure 1B, joint-angle velocities also satisfy their time-varying physical limits, the initial joint-angle velocities vector $\dot{\Theta }\left(0\right)$ equals **0**, and the time for $\dot{\Theta }\left(t\right)$ converging to zero is near to 2 s. That is, all physical limits are satisfied in the process of the self-motion task. Each joint angle as well as joint-angle velocity is not out of physical limits and does not need to be adjusted. Besides, Figure 1C depicts the value of **e**_Θ_ (joint-angle error vector **e**_Θ_ = Θ(*t*)−Θ_g_ with *e*_Θ_*i*__ being the elements of **e**_Θ_ (*i* = 1, 2, ⋯ , 6) and *t* ∈ [0, *t*_f_]), and the curves show that the joint angles gradually approach the given joint angles from initial ones over time. In addition, Figure 1F depicts end-effector position errors, which shows that the end effector keeps immobile in the practice. In specific, the maximal position error of the end effector is 1 × 10^−4^ m, and the position errors (i.e., *e*_X_ and *e*_Y_) are near zero after 2 s. As seen in Figures 1D,E, the joint angles are adjusted then gradually approach the given ones from initial joint angles, and the planar manipulator completes the task successfully.

**Figure 2:**
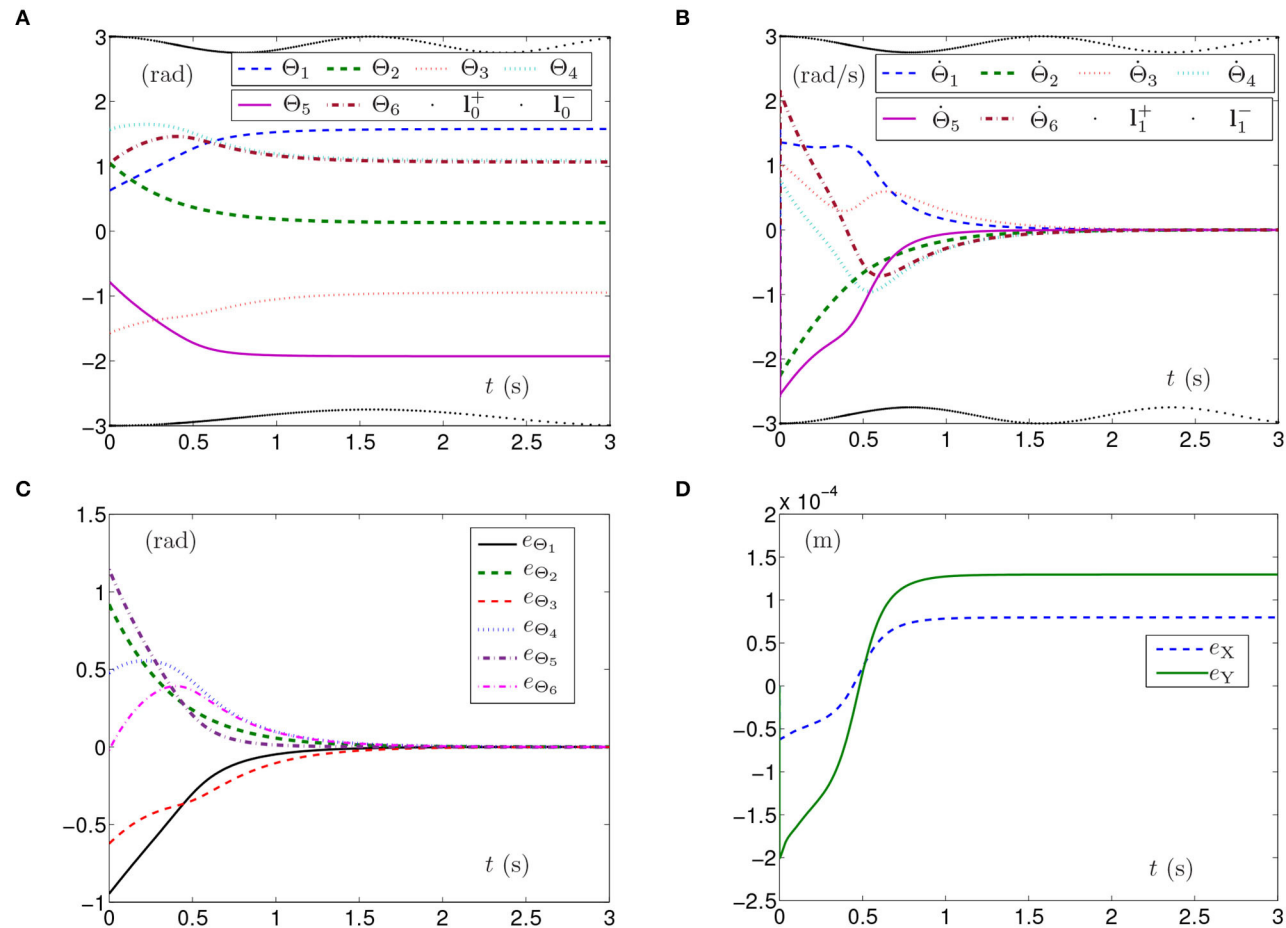
Synthesized results of the planar manipulator using SMCS-1 with time-varying physical limits satisfied in case A. **(A)** Profiles of joint angles. **(B)** Profiles of joint-angle velocities. **(C)** Profiles of joint-angle errors. **(D)** Profiles of end-effector position errors.

The simulation experiments based on the planar manipulator using SMCS-1 are carried out and the simulation results are depicted in Figure 2. The curves of joint angles with time-varying physical limits satisfied are shown in Figure 2A. However, the initial joint-angle velocities vector Θ(0) does not equal zero as presented in Figure 2B, one of which is close to time-varying physical limits of joint-angle velocities. In this case, the time for $\dot{\Theta }\left(t\right)$ converging to zero is before 2 s. The curves shown in Figure 2C indicate that each element of **e**_Θ_ converges to zero within 2 s, which also means the joint angles approach given joint angles within 2 s. As seen in Figure 2D, the maximal position error of the end effector is 1.5 × 10^−4^ m. Nevertheless, the position errors converge to some stable values but are not near zero. The joint angles also reach the given ones from initial joint angles, and the planar manipulator completes the task successfully. Due to similarity and space limitations, the corresponding pictures are omitted in the article, and the same is done in the following part.

**Figure 3:**
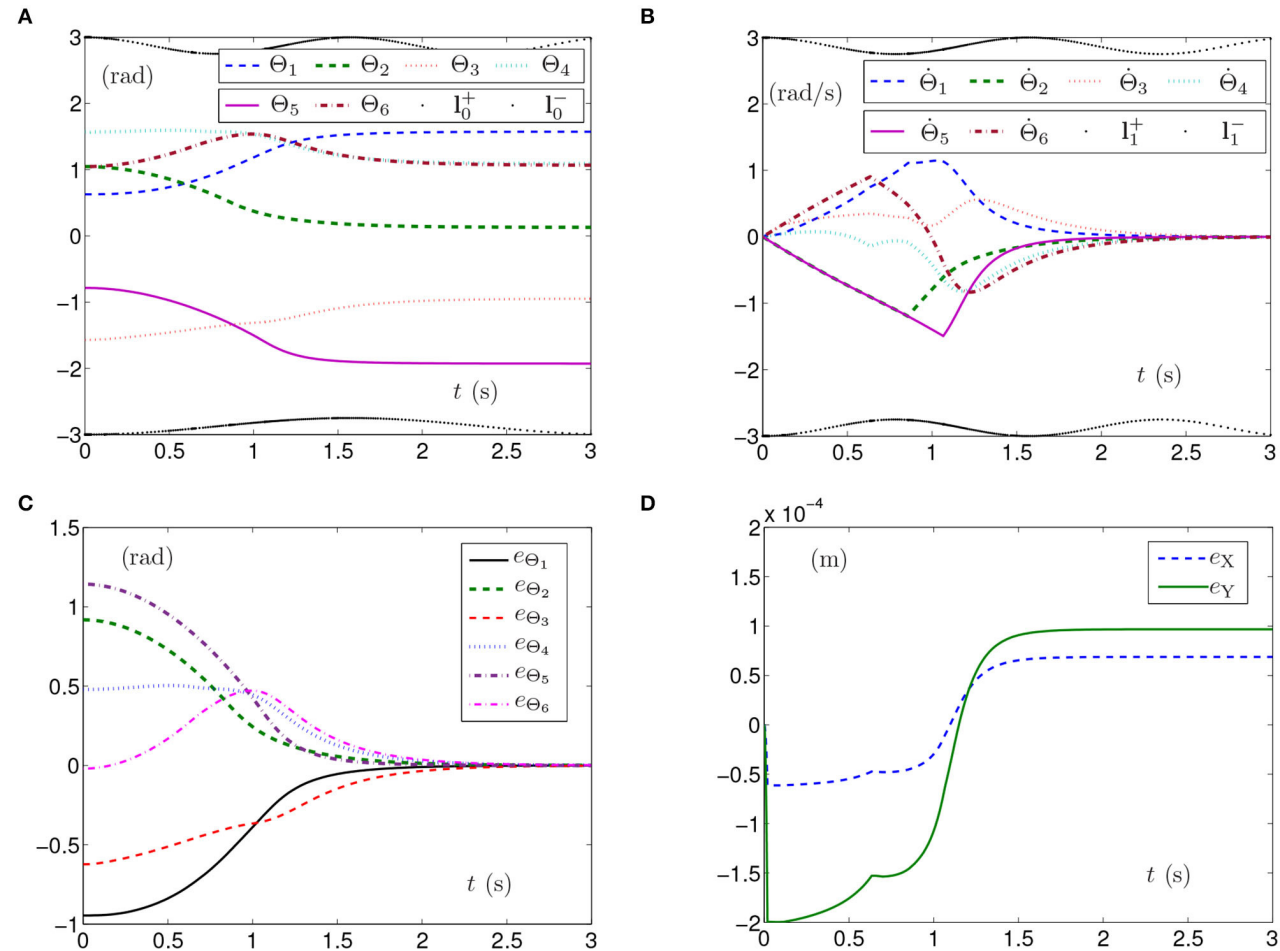
Synthesized results of the planar manipulator using SMCS-2 with time-varying physical limits satisfied in case A. **(A)** Profiles of joint angles. **(B)** Profiles of joint-angle velocities. **(C)** Profiles of joint-angle errors. **(D)** Profiles of end-effector position errors.

The simulation results synthesized by SMCS-2 are shown in Figure 3. Thereinto, Figure 3A presents the curves of joint angles with time-varying physical limits satisfied. From Figure 3B, one obtains the initial velocities of the joint angles equal to zero with the time-varying physical limits satisfied, and the time for $\dot{\Theta }\left(t\right)$ converging to zero is after 2 s, which is fractionally longer than those shown in Figures 1B, 2B. The curves shown in Figure 3C indicate that each element of **e**_Θ_ converges to zero after 2 s, which also means the joint angles approach the given joint angles after 2 s. Besides, Figure 3D depicts that the maximal position error of the end effector is 2 × 10^−4^ m with the position errors stabilized after 1.5 s.

The above three experiment results in Figures 1–3 show that when physical limits are all satisfied, the planar manipulator using SMCS-1 completes the task fastest but it does not have the zero initial velocities. On the premise that the values of initial velocities equal zero, compared with the planar manipulator using SMCS-2, the planar manipulator using SMCSvZ has a faster convergence speed and higher accuracy.

#### 5.1.2. Case B: Stringent Physical Limits

Another simulation experiment based on the planar manipulator using SMCSvZ, SMCS-1, and SMCS-2 is carried out when the region of joint-angle physical limits is not large enough. The joint-angle lower limit ${\text{l}}_{0}^{-}$ is set as[ξ, ξ, ξ, ξ, ξ, ξ]^T^ rad, where ξ = −2.1 + 0.25sin^2^(*t*). The other parameters are set the same as the above situation.

**Figure 4:**
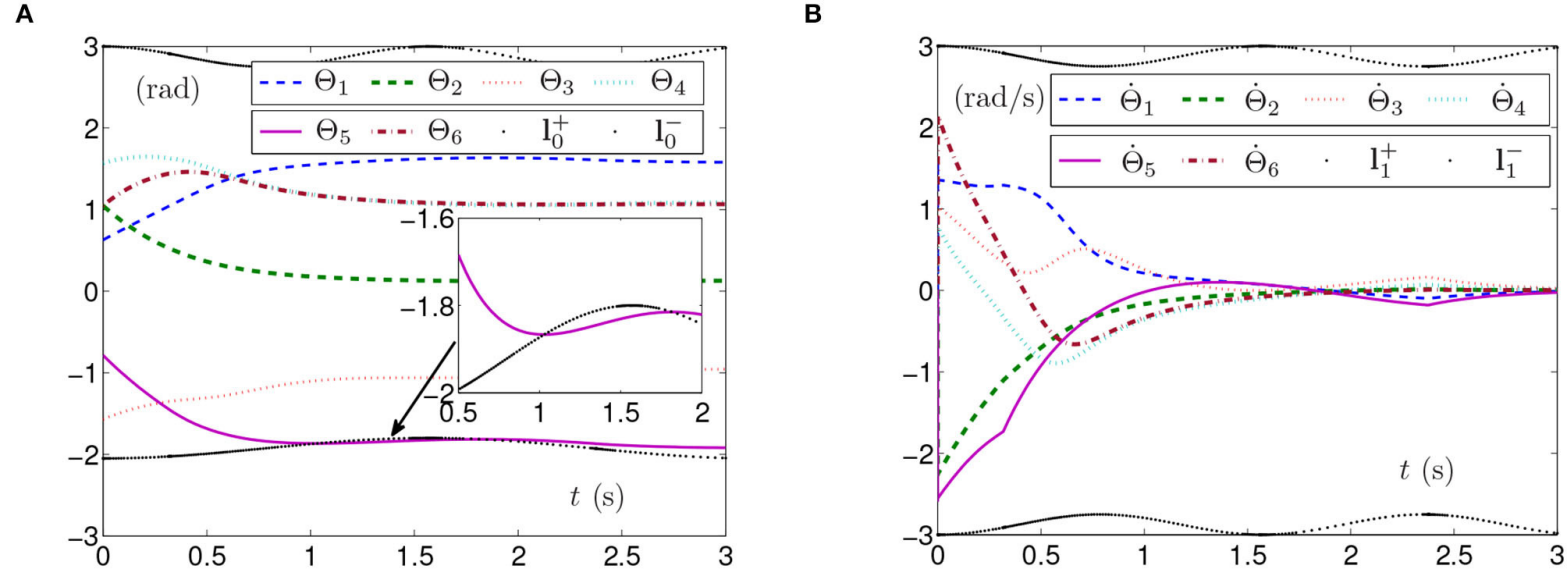
Synthesized results of the planar manipulator using SMCS-1 with time-varying physical limits unsatisfied in case B. **(A)** Profiles of joint angles. **(B)** Profiles of joint-angle velocities.

The simulation experiments based on the planar manipulator using SMCS-1 are carried out and the results are shown in Figure 4. As seen from Figure 4A, the curve of Θ_5_ exceeds the curve of time-varying lower physical limit, which means that physical limits are not satisfied and the manipulator may be damaged. The curves of the joint-angle velocities in Figure 4B present that the manipulator has adjusted, but it still cannot avoid the joint angle exceeding the physical limits.

**Figure 5:**
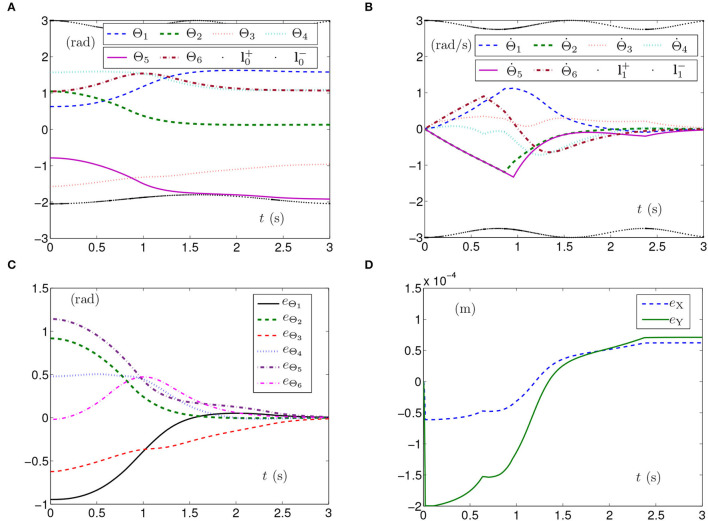
Synthesized results of the planar manipulator using SMCS-2 with time-varying physical limits verged in case B. **(A)** Profiles of joint angles. **(B)** Profiles of joint-angle velocities. **(C)** Profiles of joint-angle errors. **(D)** Profiles of end-effector position errors.

In comparison, Figure 5 depicts the simulation results synthesized by SMCS-2. To be specific, the manipulator effectively adjusts Θ_5_ to avoid exceeding the curve of the joint-angle physical limits, which means that the planar manipulator continues the self-motion task without mechanical damage in Figure 5A. As the allowable ranges of joint-angle velocities may be narrowed, the joint angles are adjusted slowly. The time for $\dot{\Theta }\left(t\right)$ converging to zero is near 3 s as depicted in Figure 5B. It takes nearly 3 s for the planar manipulator to adjust the joint angles to the given ones as depicted in Figure 5C. The maximal position error of the end effector depicted in Figure 5D is 1.5 × 10^−4^ m with the position errors converging to some stable values.

**Figure 6:**
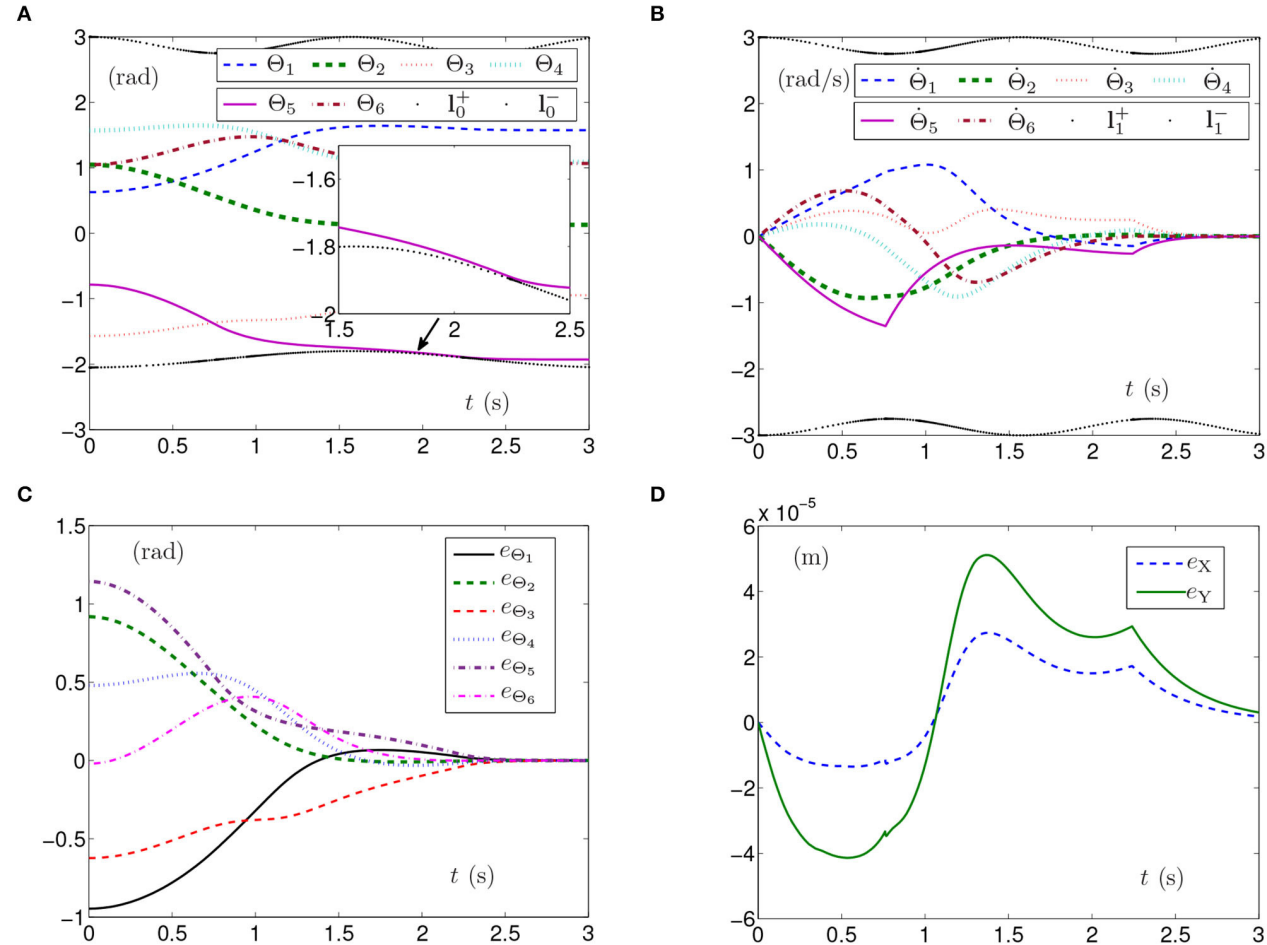
Synthesized results of the planar manipulator using SMCSvZ with time-varying physical limits verged in case B. **(A)** Profiles of joint angles. **(B)** Profiles of joint-angle velocities. **(C)** Profiles of joint-angle errors. **(D)** Profiles of end-effector position errors.

In addition to that, Figure 6 depicts the simulation results synthesized by SMCSvZ. As seen in Figure 6A, each element of Θ satisfies the joint-angle physical limits in the process of the self-motion task. When Θ_5_ is verging on its lower physical limit, the manipulator adjusts the joint-angle velocities, and thus the joint angles are correspondingly adjusted to avoid exceeding the physical limits. The time for $\dot{\Theta }\left(t\right)$ converging to zero is near 2.5 s as depicted in Figure 6B, which is less than one spent by the planar manipulator using SMCS-2. In addition, the curves shown in Figure 6C indicate that each element of **e**_Θ_ converges to zero within 2.5 s. The maximal position error of the end effector depicted in Figure 6D is 6 × 10^−4^ m.

#### 5.1.3. More Simulation Results

In this subsection, some other simulation results synthesized by the planar manipulator in case A and case B are presented. In Table 1, the data in columns 1, 3, and 5 are the values of **e**_Θ_ obtained by the planar manipulator using SMCS-1, SMCS-2, and SMCSvZ in case A, respectively. Thereinto, the maximal errors of joint angles produced by SMCSvZ and SMCS-1 are of the order of 10^−3^ m, while it is of the order of 10^−2^ m that produced by SMCS-2. In addition, the data in columns 2, 4, and 6 in Table 1 are the error values of **e**_Θ_ obtained in case B. The maximal error of joint angles produced by SMCSvZ is of the order of 10^−3^ m, while it is of the order of 10^−2^ m when produced by SMCS-1, and it is of the order of 10^−1^ m when produced by SMCS-2. In this respect, the SMCSvZ is better than the other two schemes.

**Table 1:** Values of **e** synthesized by SMCS-1, SMCS-2, and SMCSvZ in case A and case B with **e** = Θ(*t*_f_)−Θ_g_.

**Joint-angle**	**1**	**2**	**3**	**4**	**5**	**6**

	^**a**^ ${\text{e}}_{1}\times 10^{-3}$	^**a**^ ${\text{e}}_{1}^{*}\times 10^{-2}$	^**a**^ ${\text{e}}_{2}\times 10^{-2}$	^**a**^ ${\text{e}}_{2}^{*}\times 10^{-1}$	^**a**^ ${\text{e}}_{z}\times 10^{-3}$	^**a**^ ${\text{e}}_{z}^{*}\times 10^{-3}$
Θ_1_	0.4035277546	0.5782355172	0.0015447915	0.0565260232	0.2921514678	0.5103526402
Θ_2_	0.3129608608	−0.0545327541	0.0818730936	−0.0038875074	0.3244167469	0.1505453049
Θ_3_	−0.5012693542	−0.8850811496	−0.1953758157	−0.1167433302	0.3180588608	−0.2854495128
Θ_4_	0.1551459332	−0.3796040427	0.1295147141	−0.0181627340	0.2767581164	−0.1041636237
Θ_5_	−0.5392726233	0.8367928486	−0.0282762806	0.0922359849	0.1932659195	−0.3721375789
Θ_6_	0.2316018914	−0.0482526950	0.1809797644	0.0281766251	0.1004131585	−0.0027968302

^a^**e**_1_, **e**_2_, and **e**_z_ are obtained in case A, and ${\text{e}}_{1}^{*}$, ${\text{e}}_{2}^{*}$, and ${\text{e}}_{z}^{*}$ are obtained in case B with subscripts _1_, _2_, and _z_ representing SMCS-1, SMCS-2, and SMCSvZ, respectively.

**Figure 7:**
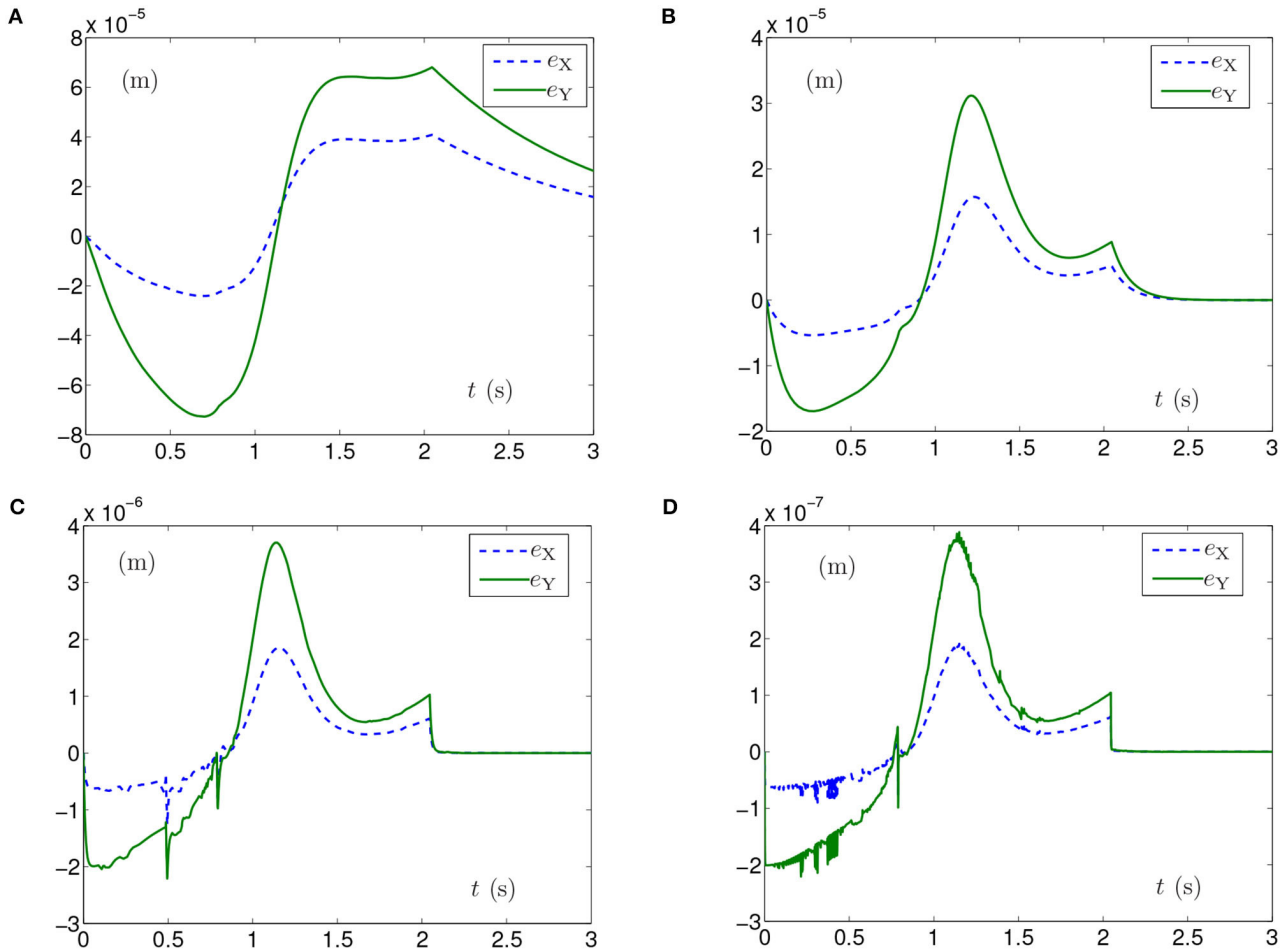
End-effector position errors of planar manipulator using SMCSvZ with time-varying physical limits verged in Case B. **(A)** With μ_2_ = 1. **(B)** With μ_2_ = 10. **(C)** With μ_2_ = 100. **(D)** With μ_2_ = 1, 000.

Generally, the parameters in the simulations influence the simulation results. For example, the position errors of the end effector reflect whether the end effector keeps motionless or not. As shown in Figures 1F, 2D, 3D, 5D, 6D, the maximal position errors are mostly of the order of 10^−4^ m when the parameter γ is set as 10^4^ in case A and case B, which meet the practical requirements. If one desires to change the precision of position error, the value of γ can be changed as Table 2 shown. For example, if γ is set as 10^5^, the 6-DoF planar manipulator using SMCSvZ completes the self-motion, and the maximal position errors are of the order of 10^−6^ m. Furthermore, to improve the precision of position error, we can also change the value of μ_2_. The simulation experiments are conducted based on the planar manipulator using SMCSvZ with different values of μ_2_ in case B, and the different results of position errors are displayed in Figure 7. As seen in Figure 7A, when μ_2_ is set as 1, the position error of the end effector does not converge within the duration of the task. When μ_2_ is set as 10, the maximal position error of the end effector depicted in Figure 7B is 4 × 10^−4^ m and its convergence time is shortened. The position errors depicted in Figures 7C,D are of the orders of 10^−5^ m and 10^−6^ m, respectively. The design parameters can be set as appropriate values according to actual requirements.

**Table 2:** Relation between position-error order and parameter γ in simulations based on planar manipulator using different control schemes in case A and case B.

		**Order 10^−6^**	**Order 10^−5^**	**Order 10^−4^**	**Order 10^−3^**
SMCSvZ	Case A	γ = 10^8^, 10^7^, 10^6^	γ = 10^5^	γ = 10^4^	γ = 10^3^
	Case B	γ = 10^8^, 10^7^, 10^6^, 10^5^	γ = 10^4^	γ = 10^3^	γ = 10^2^
SMCS-1	Case A	γ = 10^7^	γ = 10^8^, 10^6^, 10^5^	γ = 10^4^	γ = 10^3^
	Case B	γ = 10^7^	γ = 10^8^, 10^6^, 10^5^	γ = 10^4^	γ = 10^3^
SMCS-2	Case A	–	γ = 10^8^, 10^7^, 10^6^, 10^5^	γ = 10^4^	γ = 10^3^
	Case B	γ = 10^6^	γ = 10^8^, 10^7^, 10^5^	γ = 10^4^	γ = 10^3^

In summary, with the desired precision and the physical limits satisfied, the planar manipulator using SMCSvZ completes the self-motion task more effectively and efficiently compared with the ones using SMCS-1 and SMCS-2.

### 5.2. Simulations Based on PUMA560 Manipulator

To further verify the efficiency of the proposed SMCSvZ, we conduct more simulation experiments based on the PUMA560 manipulator using SMCSvZ, SMCS-1, and SMCS-2.

The task time-interval of all simulation experiments is also set as [0, 3] s. The initial joint angles are set as [0, −π/4, 0, π/2, −π/4, 0]^T^ rad, and the given joint angles are set as [0.1723, −0.9099, 0.122, 0, 0.0067, 0]^T^ rad. Specifically, each element in ${\text{l}}_{0}^{-}\left(t\right)$ is set as −3+0.25sin^2^(*t*) rad, and each element in ${\text{l}}_{0}^{+}\left(t\right)$ is set as 3−0.25sin^2^(2*t*) rad. Each element in ${\text{l}}_{1}^{+}\left(t\right)$ is set as 3−0.25sin^2^(2*t*) rad/s, and each element in ${\text{l}}_{1}^{-}\left(t\right)$ is set as −3+0.25sin^2^(2*t*) rad/s. The parameter γ = 1 × 10^5^, and other parameters are set the same as above situation.

**Figure 8:**
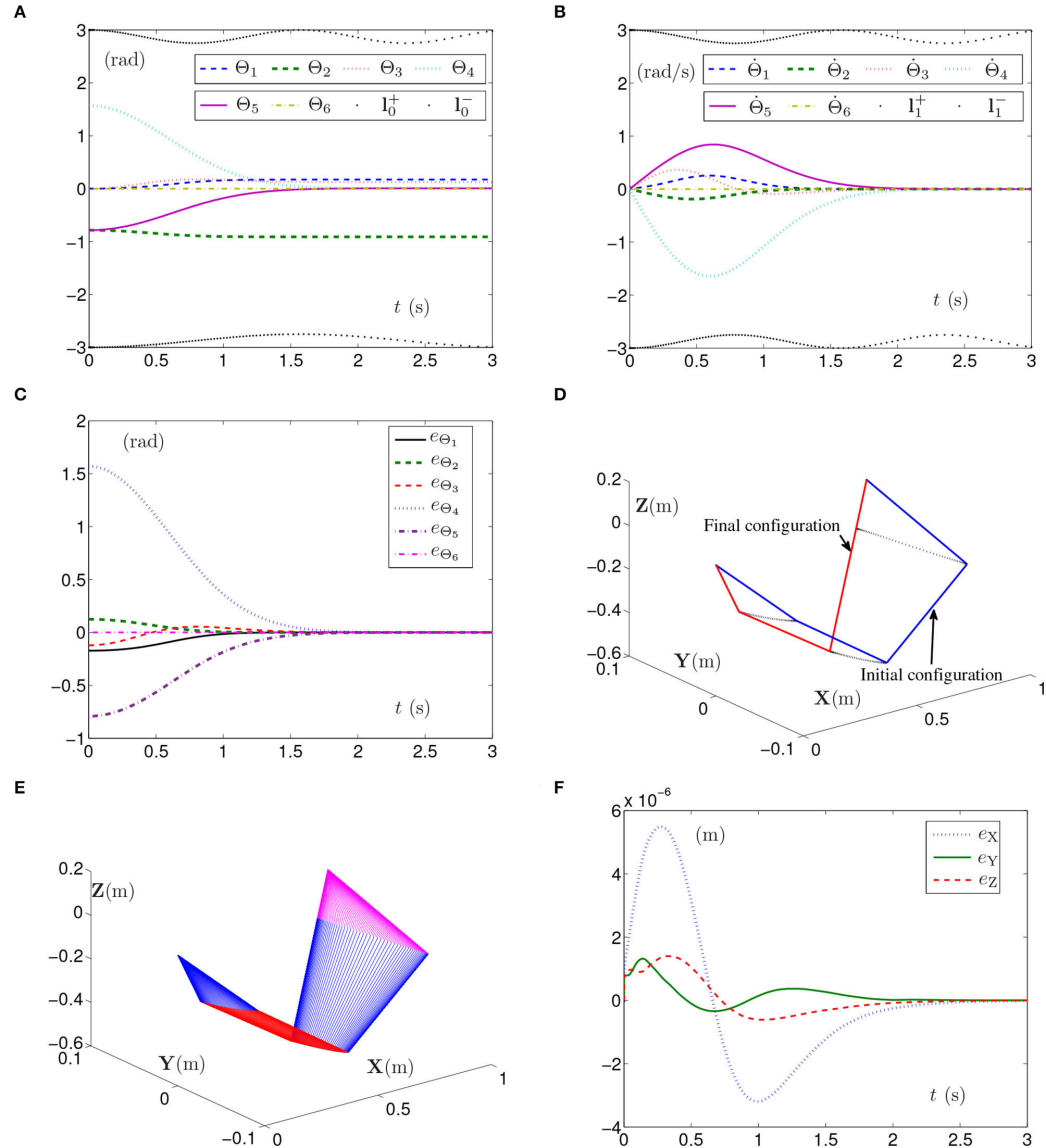
Synthesized results of the PUMA560 manipulator using SMCSvZ with time-varying physical limits satisfied. **(A)** Profiles of joint angles. **(B)** Profiles of joint-angle velocities. **(C)** Profiles of joint-angle errors. **(D)** Profiles of initial and final manipulator positions. **(E)** Profiles of the planar manipulator. **(F)** Profiles of end-effector position errors.

The simulation experiment based on the PUMA560 manipulator using SMCSvZ with the time-varying physical limits satisfied is done, and the results are displayed in Figure 8. The curves of joint angles are presented in Figure 8A. As seen in Figure 8B, the initial velocities of the joint angles equal zero, and the values of $\dot{\Theta }\left(t\right)$ converge to zero before 2 s. Besides, Figure 8C shows that the values of **e**_Θ_ also converge to zero before 2 s. In addition, Figure 8F depicts that the maximal position error of the end effector is 6 × 10^−6^ m, and the values of position errors (i.e., *e*_X_, *e*_Y_, and *e*_Z_) are close to zero over time. As seen in Figures 8D,E, the joint angles reach the given ones from initial joint angles, and the PUMA560 manipulator completes the task successfully.

**Figure 9:**
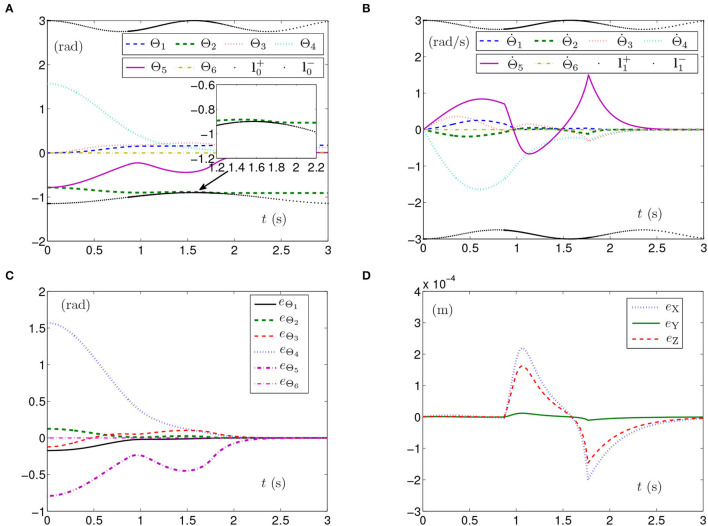
Synthesized results of PUMA560 manipulator using SMCSvZ with time-varying physical limits verged. **(A)** Profiles of joint angles. **(B)** Profiles of joint-angle velocities. **(C)** Profiles of joint-angle errors. **(D)** Profiles of end-effector position errors.

When the joint-angle lower limit ${\text{l}}_{0}^{-}$ is set as [ξ, ξ, ξ, ξ, ξ, ξ]^T^ rad with ξ = −1.15 + 0.25sin^2^(*t*), the simulation results synthesized by the PUMA560 manipulator using the SMCSvZ are shown in Figure 9. As seen in Figure 9A, the self-motion task is completed in 3 s. Apparently, the curve of Θ_2_ verges on the curve of the limit, and all physical limits are satisfied in task durations. In Figure 9B, the self-motion requirement $\dot{\Theta }\left(0\right)=0$ is satisfied, and $\dot{\Theta }\left(t\right)$ converges to **0** over time. In Figure 9C, the values of **e**_Θ_ increasingly verge on **0**. The maximal position error of the end effector is 3 × 10^−4^ m, and the values of position errors change slightly but converge to zero over time in Figure 9D, which indicates that the end effector also dynamically keeps immobile.

The simulation results synthesized by the PUMA560 manipulator using the SMCS-1 or SMCS-2 are similar to the results of the planar manipulator, which are omitted. To sum up, the PUMA560 manipulator using SMCSvZ can better meet the self-motion requirements, satisfy the time-varying physical limits, and complete the self-motion task efficiently.

## 6. Conclusion

We have proposed a refined QP-based self-motion control scheme of redundant robot manipulators with time-varying joint limits and zero initial joint-angle velocities satisfied *via* the ZNDE approach in the paper. The proposed scheme has been composed of a ZNDE equation constraint and a bound ZNDE inequation constraint. Compared with two previous SMCSs, we have theoretically analyzed the proposed SMCSvZ that well meets the self-motion requirements, then applied it to control the redundant robot manipulators for the self-motion task. The simulation experiments have been conducted based on the 6-DoF planar manipulator in two different cases. By comparing with the simulation results produced by the redundant robot manipulators using SMCS-1, SMCS-2, and SMCSvZ, the proposed SMCSvZ has shown its effectiveness, superiority, and practicability. Besides, the simulation results produced by the PUMA560 manipulator using SMCSvZ in two different cases have been obtained, and they have also verified the feasibility and correctness of the SMCSvZ. Based on ZNDE, more kinds of time-varying problems would be simplified and solved in future studies. Besides, the scheme established in the article is continuous-time and is not convenient for hardware implementation, and thus, the design and development of a discrete-time scheme could be one future research direction.

## Data Availability Statement

The raw data supporting the conclusions of this article will be made available by the authors, without undue reservation.

## Author Contributions

ZT and YZ proposed the scheme and wrote the manuscript. ZT designed and carried out experiments. Both authors contributed to the article and approved the submitted version.

## Funding

This study was aided by the National Natural Science Foundation of China (61976230), the project supported by Guangdong Province Universities and Colleges Pearl River Scholar Funded Scheme (2018), the Key-Area Research and Development Program of Guangzhou (202007030004), the Research Fund Program of Guangdong Key Laboratory of Modern Control Technology (2017B030314165), the Shenzhen Science and Technology Plan Project (JCYJ20170818154936083), and also the project supported by Hunan Education Department (19C1529).

## Conflict of Interest

The authors declare that the research was conducted in the absence of any commercial or financial relationships that could be construed as a potential conflict of interest. The reviewer DC declared a shared affiliation with the authors ZT, YZ to the handling editor at the time of review.

## Publisher's Note

All claims expressed in this article are solely those of the authors and do not necessarily represent those of their affiliated organizations, or those of the publisher, the editors and the reviewers. Any product that may be evaluated in this article, or claim that may be made by its manufacturer, is not guaranteed or endorsed by the publisher.
